# Editorial: Developmental delay and intellectual disability

**DOI:** 10.3389/fgene.2022.934815

**Published:** 2022-09-08

**Authors:** Santasree Banerjee, Anjana Munshi, Chen Li, Muhammad Ayub

**Affiliations:** ^1^ Department of Genetics, College of Basic Medical Sciences, Jilin University, Changchun, China; ^2^ Department of Human Genetics and Molecular Medicine, School of Health Sciences, Central University of Punjab, Bathinda, India; ^3^ School of Medicine, Graduate School, Zhejiang University, Hangzhou, China; ^4^ Zhejiang Provincial Key Laboratory of Genetic and Developmental Disorders, Zhejiang University School of Medicine, Hangzhou, China; ^5^ Department of Psychiatry, Queens University, Kingston, Ontario, Canada

**Keywords:** developmental delay, intellectual disabilities, chromosome microarray, whole exome sequencing, novel mutations

Intellectual Disability (ID) is characterised by impaired intellectual and adaptive function that starts during the developmental period (Global Research on Developmental Disabilities Collaborators, 2022). It is a lifelong condition and is among the most common neurodevelopmental disorders (Global Research on Developmental Disabilities Collaborators, 2018). Developmental Delay (DD) is a broad term that applies when one or more areas of a child’s development are delayed (Global Research on Developmental Disabilities Collaborators, 2018). A disease-causing variant is identified in about half of individuals with DD and ID. Both copy number variants (CNVs) and single nucleotide variants (SNVs) are implicated as major causes of DD and ID (de Ligt et al., 2012; Olusanya and Nair, 2019); more than 130 rare CNVs and SNVs in more than 600 genes have been reported to be associated with this disease spectrum (de Ligt et al., 2012). Previous reports have shown that DD and ID can follow autosomal dominant, autosomal recessive, or X-linked modes of inheritance, as well as making a substantial contribution from *de novo* variants to the molecular aetiology (de Ligt et al., 2012; Black and Lawn, 2018).

Further research into the identification of candidate causal genes will create opportunities to study the molecular mechanisms underlying the disease phenotype and will enable clinicians to make timely and accurate clinical diagnoses (American Psychiatric Association, 2013; Heslop et al., 2014; Black and Lawn, 2018). This will make it possible to group cohorts of cases with similar genetic aetiology and refine their phenotypes including the prognosis and course of the disease. Genetic diagnosis will allow mutational screening in families and make prevention possible through genetic counselling, prenatal, or pre-implantation diagnosis (Tatja et al., 2021).

According to the American Association on Intellectual and Developmental Disabilities, affected individuals with global developmental delay are characterised by a significant delay in the development of motor skills, speech, and cognition, while ID involves deficits in both intellectual function (learning, reasoning, and problem-solving skills) and adaptive function (communication, conceptual, social and practical skills) among children aged 5 years or younger. The global prevalence of DD is 1–3% among children aged 5 years or younger (de Ligt et al., 2012; Olusanya and Nair, 2019). The prevalence of ID is 2.7% and 2.17% among children and adults respectively. In addition, DD patients with *de novo* mutations have a prevalence rate of 1 in 213 to 1 in 448 live-births (American Psychiatric Association, 2013; Heslop et al., 2014; Black and Lawn, 2018; Tatja et al., 2021).

Advances in variant detection technology have evolved in recent years, leading to accelerated causal gene discovery and understanding of genomic lesions in ID/DD cohorts. After preliminary confirmation of the disease through clinical, laboratory and radiological examination, clinicians can order further diagnostic testing, which is influenced by initial findings and availability and access to resources. The range of potential lines of inquiry include but are not limited to: karyotyping to identify gross chromosomal abnormalities; chromosome microarray (CMA) to identify deletions, duplications, loss of heterozygosity, and aneuploidy; and genomic sequencing (target-based sequencing of a gene panel, clinical whole exome sequencing or whole genome sequencing) to identify disease-causing variants (de Ligt et al., 2012; Mefford et al., 2012).

This Research Topic features new causal genes for DD and ID, advances in the mechanistic understanding of previously reported DD and ID genes, and potential therapeutic applications. We received 40 submissions and accepted 21 papers after rigorous peer review (Cerminara et al.; Chen et al.; Deng et al.; Fan et al.; García-Ortiz et al.; Li et al.; Servetti et al.; Smetana et al.; Tao et al.; Wan et al.; Wang et al.; Xiang et al.; Zarate et al.; Zhang et al.; Ali Alghamdi et al.; Binquet et al.; Ha et al.; Li et al.; Rong et al.; Yue et al.; Zhao et al.).

Four publications are based on chromosomal aberrations associated with DD and ID. Deng et al. reported two new cases of chromosome 12q14 deletions and reviewed the published literature (Deng et al.). The authors delineate the genotype-phenotype correlation for 12q interstitial deletions and discuss likely causative genes. Wang et al. reported familial translocation t (2; 4) (q37.3; p16.3) (Wang et al.). They describe a range of complex phenotypes associated with these chromosomal abnormalities. This paper highlights the value of studying extended families for intrafamilial variation in genotypes and associated phenotypes. Smetana et al. reported a case of Xq22.3 deletion associated with Alport syndrome with intellectual disability (ATS-ID, AMME complex; OMIM #300194) with genotype phenotype correlation (Smetana et al.). The deletion is larger in size compared with previously reported cases and includes two additional genes. This may explain a broader phenotype with additional features in the proband. Fan et al. reported a case with deletion of chromosome 7q35-7q36.3, which causes congenital brain dysplasia, DD and ID (Fan et al.). Servetti et al. used an integrated framework to analyse 12 cases of neurodevelopmental disorders with complex phenotypes and suggested that those cases can be explained by multiple mechanisms, including additive effects of multiple CNVs, involving known neurodevelopmental disorder genes and novel candidate genes (Servetti et al.). One study leveraged a consanguineous family with four affected individuals to identify a likely pathogenic homozygous variant in *OSGEP* (Ali Alghamdi et al.). Detailed phenotyping and proteomic analysis are a valuable part of this study (Ali Alghamdi et al.).

Three studies are based on the evaluation of multiple cases. Wan et al. reported six new variants in seven cases and provided phenotype descriptions for MEF2C haploinsufficiency syndrome (MCHS) (Wan et al.). Xiang et al. used whole exome sequencing to investigate 17 patients with unexplained DD and/or ID (Xiang et al.). They used the whole exome data for analysis of CNV, SNV and indels. Seven affected individuals carried a single nucleotide variant or an indel that explained the disease, and three cases carried a disease-associated CNV. Zarate et al. reported two cases of SATB2-Associated Syndrome (Zarate et al.). The clinical symptoms overlapped with mitochondrial disease presentation. The authors recommended considering exome sequencing in suspected cases of mitochondrial disease.

Binquet et al. reported a study that compares the diagnostic yield of trio sequencing in 1,275 cases with the current strategy for fragile X diagnostics, which involves microarray and panel sequencing for 44 genes (Binquet et al.). This is an indication of new avenues in the field that are moving toward reduced costs of sequencing. Garcia-Ortiz et al. examined methylation in blood samples of patients with autism spectrum disorder (ASD) (García-Ortiz et al.). In a case report, Cerminara et al. reported an affected individual with a complex phenotype including ASD and its association with a maternally inherited X-linked missense variant in *HUWE1* and a *de novo* stop variant in *TPH2* (Cerminara et al.).

In a study by Chen et al., maternal uniparental disomy resulted in a homozygous variant in *CNTN2* in an affected individual with unrelated parents (Chen et al.). The predominant phenotype was a focal epilepsy. This gene has been implicated previously in autosomal recessive focal seizures. Tao et al. reported a case of complete maternal uniparental disomy of chromosome 2 with a rare *UNC80* c.5609-4G>A intronic variant in a Chinese patient with infantile hypotonia, psychomotor retardation and facial dysmorphology (Tao et al.). Disomy resulted in a homozygous mutation. These two reports reinforce the paradigm of parental disomy causing homozygous disease in outbred populations (Chen et al.; Tao et al.). Two additional papers reported compound heterozygous mutations causing recessive disorders (Yue et al. and Ha et al.) in outbred populations (Ha et al.; Yue et al.).

Li et al. described a case of mosaic Turner syndrome 45, X [56.5%]/46, X, del(Y) (q12) [43.5%] (Li et al.). They used different techniques including FISH and low pass whole genome sequencing to identify the genetic changes. Zhang et al. identified a pathogenic variant in *GRIN1* in a single case and performed further experiments to study the pharmacological impact of the DNA change and rescue mechanisms (Zhang et al.). Zhao et al. described a nonsense variant in *ZNF462* associated with Weiss-Kruszka syndrome-like manifestations (Zhao et al.). Li et al. identified a novel homozygous splice-site variant in a female child with Cohen syndrome (Li et al.). Rong et al. reported a milder phenotype produced by a novel mutation in *SEPSECS* (Rong et al.).

In conclusion, this special issue reports on both CNVs and SNVs associated with DD and ID. In the future, more refined cellular and molecular studies are required to understand the disease mechanisms (Bowling et al., 2017; Karin et al., 2019; Manickam et al., 2021). This collection emphasises the significance of genomic sequencing technology for molecular genetic diagnostics in ID/DD cohorts, thus laying the groundwork for a future Research Topic focused on studies that will identify new candidate genes and disease-causing variants, which in turn will allow us to develop novel therapeutic interventions.
